# Do Cancer Patients Present a Different Phenotype of Atrial Fibrillation or a Different Arrhythmia Management?

**DOI:** 10.3390/biomedicines13112700

**Published:** 2025-11-03

**Authors:** Maja Hawryszko, Grzegorz Sławiński, Weronika Pusz-Bulas, Mikołaj Młyński, Kalina Wiśniewska, Patryk Macuk, Aleksandra Liżewska-Springer, Ludmiła Daniłowicz-Szymanowicz, Ewa Lewicka

**Affiliations:** Department of Cardiology and Electrotherapy, Faculty of Medicine, Medical University of Gdańsk, 80-214 Gdańsk, Poland; maja.klimkiewicz@gmail.com (M.H.); werkapusz@gmail.com (W.P.-B.); mikolaj.mlynski@gmail.com (M.M.); kalina.wisniewska@gumed.edu.pl (K.W.); patryk.macuk@gumed.edu.pl (P.M.); ollizz@gumed.edu.pl (A.L.-S.); ludwik@gumed.edu.pl (L.D.-S.); elew@gumed.edu.pl (E.L.)

**Keywords:** arrhythmias, atrial fibrillation, cancer, cardio-oncology, echocardiography

## Abstract

**Background/Objectives**: Along with cardiovascular disease, cancer is the leading cause of death worldwide. The aim of the study was to compare the clinical and echocardiographic characteristics of atrial fibrillation (AF) among patients with active cancer (study group) and those without cancer (control group). **Methods**: This retrospective study included patients diagnosed with both AF and active cancer referred for consultation to the Cardiac Arrhythmias Outpatient Clinic at our institution in 2022. They were matched in a 1:1 ratio, by age and sex to patients with AF without cancer. The matching criteria for the study and control groups were limited to age and gender. Variables such as comorbidities, atrial fibrillation duration, tumor stage, and treatment were not included in the matching criteria. **Results**: We examined 216 patients, 57.4% of whom were men. There were 110 patients in the study group with a mean age of 70 (10) years. Several parameters in the study group attracted our particular attention: N-terminal pro-B-type natriuretic peptide (NT-proBNP) was lower (703 pg/mL vs. 1549 pg/mL, *p* = 0.01), echocardiography revealed smaller left atrial size (43 (6) mm vs. 47 (7) mm, *p* = 0.015), left ventricular (LV) diastolic dimension (49 mm vs. 52 mm, *p* = 0.009) and better LV systolic function expressed as LV ejection fraction (54% (9%) vs. 51% (12%), *p* = 0.025), and global longitudinal strain (−16% (4%) vs. −13% (3%), *p* = 0.016). Antiarrhythmic pharmacotherapy was used less frequently in the study group, which may also have been due to physicians’ reluctance to treat AF aggressively in a more morbid population. **Conclusions**: Patients with cancer and atrial fibrillation may have a different clinical profile, suggesting a different pathophysiology. Understanding these differences may help reduce the incidence of AF and improve patient outcomes and prognosis during cancer treatment.

## 1. Introduction

Along with cardiovascular diseases, cancer is one of the leading causes of death in both men and women worldwide. The prognosis of cancer has improved in recent decades; however, cancer treatment is associated with many side effects, including cardiotoxicity. Chemotherapy regimens have the potential to modify membrane ion channel activity and calcium balance within cardiomyocytes, consequently influencing their electrophysiological properties. This effect is observed across various classes of drugs, including anthracyclines, taxanes, antimetabolites, and alkylating agents. Tyrosine kinase inhibitors can also affect ion channels and cellular signaling pathways [1,2]. These changes may contribute to ischemia or left ventricular (LV) systolic dysfunction, which further increases the risk of arrhythmia occurrence [1]. The risk is also increased by the coexistence of metabolic disorders, increased tone of the sympathetic nervous system, and cancer-induced generalized inflammation [1,2,3,4].

Among cardiac arrhythmias, atrial fibrillation (AF) is the most prevalent arrhythmia in the general population (2–4%) and its prevalence increases with age [5,6,7,8]. It is more common in Caucasian males, and other contributing factors include arterial hypertension, diabetes, hypothyroidism, ischemic heart disease, heart failure, valvular heart disease, obesity, and obstructive sleep apnea [5,6].

The higher incidence of AF in cancer patients may be a result of the increased inflammation and oxidative stress caused by the cancer itself, as well as its various treatments. Atrial fibrosis, inflammation, and oxidative stress are key mechanisms that collectively promote the pro-arrhythmic and pro-thrombotic environment characteristic of AF. In addition, increase in cancer survivors may contribute to the increase in the incidence of AF in these patients [6,9,10].

The aim of the study was to compare clinical characteristics and echocardiographic parameters of AF patients with active cancer (study group) and AF patients without cancer (control group).

## 2. Materials and Methods

### 2.1. Patients

All patients from the Cardiac Arrhythmias Outpatient Clinic at our institution diagnosed with AF and active cancer (treated in the past 6 months or currently), selected from 1 January 2022 to 31 December 2022, were included in the retrospective, single-center study. These patients were matched in a 1:1 ratio on age and sex with controls who were cancer-free patients from the same outpatient clinic with diagnosis of AF. All patients with persistent and permanent atrial fibrillation at the time of inclusion in the study were circulatory stable, without signs of circulatory decompensation, and without the need for hospitalization for this reason in the last six months, which indirectly confirmed good control of ventricular rate. The statistical analysis included demographic data, comorbidities, medications used, laboratory tests results, and echocardiographic parameters. Variables such as comorbidities, atrial fibrillation duration, tumor stage, and treatment were not included in the matching criteria.

The cardiovascular risk factors (CVRF) included smoking (active or former), arterial hypertension, diabetes, obesity, and hypercholesterolemia. Hypercholesterolemia was defined as low-density lipoprotein (LDL) cholesterol > 130 mg/dL or statin use. In relation to anticoagulant treatment, the frequency of apixaban, rivaroxaban, dabigatran, vitamin K antagonists (VKA), and low-molecular-weight heparin (LMWH) use was assessed.

Bleeding has been reported during anticoagulant therapy. Major bleeding was defined according to The International Society on Thrombosis and Haemostasis (ISTH) definition [11,12] as bleeding resulting in a reduction in hemoglobin concentration by ≥2 g/dL, requiring transfusion of ≥2 units of packed red blood cells or whole blood, occurring in a critical location (i.e., intracranial, intraspinal, intraocular, intra-articular, retroperitoneal, pericardial), or causing the patient’s death.

Clinically relevant non-major bleeding (CRNMB) was defined [11,12] as clinically overt, not major bleeding, and requiring any medical intervention, surgical treatment or hospital admission.

### 2.2. Statistical Analysis

A *p*-value of less than 0.05 was considered statistically significant for all comparisons and calculations. Continuous variables were expressed as the mean ± SD if normally distributed or median if not normally distributed. In the case of continuous variables, normal distribution was tested by using the 1-sample Kolmogorov–Smirnov test. Categorical data were expressed as numbers and percentages. Continuous variables were compared using independent-sample parametric (unpaired Student *t*) or nonparametric (Mann–Whitney *U*, Kruskal–Wallis test by ranks) tests. Categorical variables were compared using the chi-square test or the Fisher exact test when appropriate. Correlations between selected quantitative variables were assessed using Spearman’s rank correlation test. The data were analyzed using the STATISTICA 13 software. The study was approved by the Bioethical Committee at the Medical University of Gdansk (KB/315/2024).

## 3. Results

### 3.1. Clinical Features

**Figure 1:**
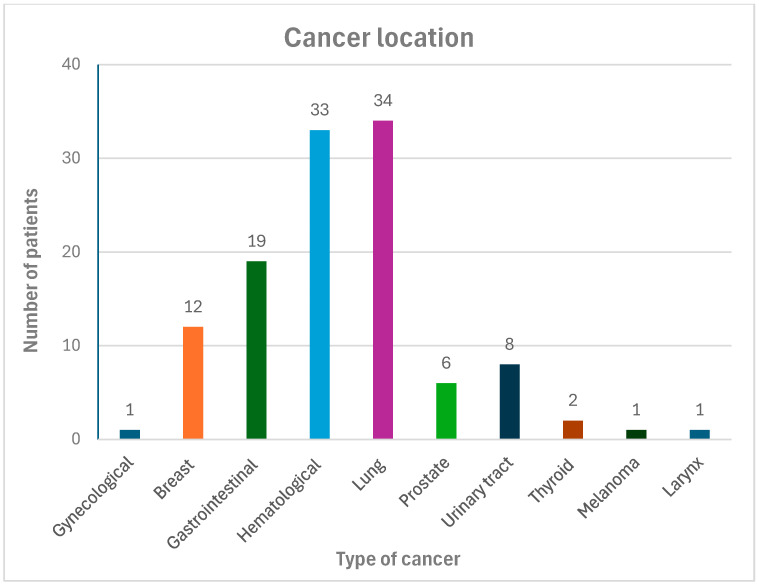
Cancer location in the study group.

The study included 216 patients, and 124 (57.4%) were men. The study group consisted of 110 patients in the mean age of 70 (10) years. The study group had a higher Charlson Comorbidity Index (CCI): median 4 vs. 2 points (*p* < 0.001). The most common cancers in the study group were lung cancer (n = 34; 29%) and hematological malignancies (n = 33; 28%), as shown in Figure 1. Patients in the study group were significantly more likely to use LMWH anticoagulant therapy (17 patients vs. 1 patient, *p* = 0.001), while 61.8% of patients were taking direct oral anticoagulant (DOAC) compared to 70.1% of patients in the control group which was not significantly different. Similarly, there were no differences between the groups in the incidence of left atrial appendage (LAA) thrombi presence (1 vs. 5 patients, *p* = 0.09) and the incidence of major bleedings (9 vs. 4 patients, *p* = 0.168), which were not statistically significant.

There were no differences in such CVRFs as arterial hypertension, coronary artery disease, and diabetes; however, chronic obstructive pulmonary disease (COPD) was more common in the study group, as was the number of active or former smokers (Table 1). In both groups common risk factors for AF (arterial hypertension, coronary artery disease, and diabetes) were present, as reported in other studies; however, chronic obstructive pulmonary disease was more common in the study group, as was the number of active or former smokers.

Patients in the study group were less often undergoing electrical cardioversion (21 vs. 37 patients, *p* = 0.01) and pulmonary vein isolation (3 vs. 23 patients, *p* < 0.001). Antiarrhythmic pharmacotherapy (excluding beta-blockers) was used less frequently in the study group (16 vs. 47 patients, *p* < 0.001). Nevertheless, there was no difference (30 vs. 25 patients, *p* = 0.53) in the number of patients with permanent AF. Patients from the study group, compared to controls, had lower median serum concentration of N-terminal pro-B-type natriuretic peptide (NT-proBNP): 703 pg/mL vs. 1549 pg/mL (*p* = 0.010), with no difference in the serum B-type natriuretic peptide (BNP) level, and creatinine concentration. Demographic and clinical characteristics of both groups are shown in Table 1.

### 3.2. Echocardiographic Parameters

Patients in the study group compared to controls showed less enlarged left atrium (LA), both when assessing the LA antero- posterior diameter (43 (6) mm vs. 47 (7) mm, *p* = 0.015) and LA volume (90 (30) mL vs. 113 (66) mL, *p* = 0.04). Also, taking into account the right atrial area, its enlargement was found less often in the study group compared to the control group (15% vs. 31%, *p* = 0.004). The diastolic LV diameter was also lower in the study group (49 mm vs. 52 mm, *p* = 0.009). There were significant differences in the LV systolic function between the groups. Patients from the study group showed better LV systolic function, both when comparing the LV ejection fraction (LVEF): 54% (9%) vs. 51% (12%), (*p* = 0.025) and LV global longitudinal strain (LV GLS): −16% (4%) vs. −13% (3%), *p* = 0.016). Echocardiographic findings are shown in Table 2.

## 4. Discussion

Significant differences between cancer and cancer-free patients with AF may indicate a different pathophysiology of this arrhythmia and its transient character in cancer patients. It draws attention that there were no differences in the presence of common risk factors for AF—arterial hypertension, coronary artery disease, and diabetes, as reported in other studies [13,14]. Also, the number of patients with ≥2 CVRFs or ≥3 CVRFs did not differ between the groups. In cancer patients, only COPD was more common, as was nicotine addiction.

Moreover, they showed significantly higher CCI, but it was predictable due to the burden of oncological disease. The median CCI was 4 points, and a value of ≥4 is considered high. In patients without cancer, the median CCI was 2 points. Jung et al. showed that the same CCI value in a patient with AF indicates a higher risk of death from any cause, stroke, major bleeding, and a higher risk of hospitalization due to heart failure when compared to a patient without AF [15]. The same applies to increased CCI values in cancer patients regardless of the presence of arrhythmia [15,16]. Interestingly, despite higher CCI, cancer patients did not show worse LV systolic function or higher concentrations of natriuretic peptides. The NT-proBNP level was even lower in these patients. In addition, antiarrhythmic medications were used significantly less frequently in cancer patients: 4.5% vs. 44% patients (*p* < 0.001), but despite this, the percentage of patients with permanent AF didn’t differ between the groups: 27.3% vs. 23.4%, (*p* = 0.53). All this may be due to different pathophysiology of AF in cancer population. Approximately 70% of atrial fibrillation (AF) cases are associated with underlying heart disease, while the remaining 30% occur without an identifiable structural cause. Atrial fibrosis, which features in the structural remodeling associated with AF, is a common pathological result of various conditions, including heart failure, obesity, and hypertension. These conditions increase the risk of developing AF [17]. Key mechanisms of AF in cancer patients may involve inflammation and oxidative stress caused by the cancer or its treatment. The buildup of fibrous collagen leads to fibrosis from these conditions. This collagen may act as a reparative response to replace damaged heart tissue, or it may occur as reactive fibrosis, resulting in interstitial tissue expansion [17]. Recent research has emphasized the role of pericardial adipose tissue in promoting atrial fibrosis and the significance of genetic predisposition.

Anticoagulant therapy poses a significant challenge for cancer patients with AF and a high CCI. Recent cardio-oncology guidelines recommend DOAC as the first-line therapy, although they have not been subjected to any specific randomized clinical trial for AF in patients with cancer. Post hoc analyses of studies involving direct factor Xa inhibitors in AF patients (ROCKET AF, ARISTOTLE, ENGAGE AF-TIMI trial) and observational data indicate that DOAC are not only safer in terms of bleeding but also at least as effective as VKA in patients with active cancer [5,18].

The majority of cancer patients (61.8%) were treated by DOAC, compared to 70.1% in the control group. This shows that DOAC may be a viable and well-tolerated anticoagulant treatment option in cancer patients, as was reported also by other authors [19]. Importantly, the majority of patients were those with cancers of a “bleeding potential”, as 52% of patients had lung, gastrointestinal or urinary tract cancer, and another 28% of patients had hematological malignancies. Importantly, major bleeding was rare and there was no difference between the groups.

Cancer patients were significantly less likely to undergo electrical cardioversion and pulmonary vein isolation for AF. Menezes Fernandes et al. also reported that only 8.4% of patients with AF and a recent diagnosis of cancer underwent outpatient electrical cardioversion compared to 63.8% of cancer-free AF patients, and the referral for AF or atrial flutter ablation was significantly lower in cancer patients: 1.9% vs. 11.6% (*p* = 0.027) [19]. Data in the literature on performing catheter ablation (CA) in patients with active cancer are scarce. However, if the patient has already completed oncological treatment, or such treatment will be used in the long term and survival is expected to be >12 months in good general condition, CA should be considered, especially in symptomatic patients, as this treatment can significantly improve their quality of life. Similar requirements are used and widely accepted considering ICD implantation [20]. Giustozzi et al. examined the safety of CA for the treatment of nonvalvular AF in cancer survivors, and found that clinically significant bleeding was more common in cancer survivors than in patients without cancer [21]. On the contrary, one retrospective study showed that CA for the treatment of AF is both safe and effective in patients with a history of cancer, including those exposed to potentially cardiotoxic oncological treatments. This study included 251 cancer patients, also those who were undergoing anticancer therapy in the CA period. During the 12-month follow-up after CA, there was no difference in the percentage of patients free from AF between those with and those without cancer: 83.3% vs. 72.5% (*p* = 0.28). There were also no differences in the frequency of complications over the 3-month period after CA [22].

We find interesting results of comparison of echocardiographic parameters between the groups. Cancer patients showed less LA enlargement, and they were also less likely to have right atrial enlargement. We hypothesize that AF patients with cancer showed smaller atria due to a different pathomechanism of this arrhythmia which probably involves the pro-inflammatory cytokines and the exacerbated inflammatory response to cancer treatment. In addition, AF episodes in cancer patients may be shorter in duration and tend to self-limit with the cessation of precipitating factors (chemotherapy administration, vomiting, diarrhea, pain). This may explain postponed negative remodeling of the LA. It is consistent with Farkowski et al. finding, where based on atrial mapping during CA procedure, more than 70% of patients with cancer and AF had no significant LA scarring (fibrosis) [23]. In patients with nonvalvular AF, the left atrium is usually enlarged. Several studies confirmed this observation [5]. This enlargement is closely related to the occurrence of complications such as thromboembolism heart failure, and mitral regurgitation associated with further LA enlargement [5,24]. There is a lack of studies with echocardiographic examination in relation to AF in cancer patients, as well as data on AF burden in these population. Our study is a preliminary step for future prospective research on this issue.

In our study, we observed lower rates of antiarrhythmic therapy, cardioversion, and ablation in patients with cancer. However, it is unclear whether these differences reflect differences in symptom severity, higher morbidity, patient preferences, or clinical contraindications. We suspect, however, that physicians are less involved in optimizing the treatment of AF in patients with cancer.

### Limitations

The study and control groups were only matched for age and gender. Comorbidities, duration of atrial fibrillation, and atrial size prior to treatment were not factors in the matching process. Therefore, we were unable to refer to the additional burdens associated with specific subtypes and compare them with each other. Unfortunately, in both groups, we had no data on the AF history duration. The duration of persistent and permanent AF is a critical factor, as different durations can have significant effects on atrial size and ventricular function. The ventricular rate in persistent and permanent AF is also a critical factor, as different rates can have significant effects on atrial size and ventricular function. Cancer patients were at different stages of the disease and undergoing various treatment methods. In most patients, we did not have any data on chemotherapy regimens and radiotherapy methods, and these factors were not included in the statistical analysis. Therefore, although the obtained results can be interesting, we are critical of them and recognize that they require confirmation in larger groups of patients.

## 5. Conclusions

Patients with cancer who are diagnosed with atrial fibrillation may exhibit a different clinical phenotype than patients with atrial fibrillation who do not have cancer. This may indicate a different pathophysiology of this arrhythmia in cancer patients. If the factors related to the cancer itself and its treatment are dominant for the occurrence of AF, intensive control of comorbidities and cardiovascular risk factors can result in reduction of arrhythmia burden in these patients. This is of significant clinical importance because the occurrence of AF during cancer treatment may affect the prognosis in cancer patients. Undoubtedly, cancer is not an absolute contraindication to catheter ablation in the treatment of AF, and it seems urgent to formulate recommendations on this subject for this group of patients in the near future.

## Figures and Tables

**Table 1:** Demographic and clinical characteristics of the study and control group.

Variable	All Patients n = 217	Cancer Patients n = 110	Control Group n = 107	*p* Value
Age (years)	70 (10)	70 (9)	70 (10)	0.855
Males, n (%)	124 (57.4)	63 (57.3)	61 (57)	0.964
**BMI (kg/m^2^)**	28 (25–31)	27 (25–30)	29 (26–32)	**0.028**
Systolic blood pressure (mmHg)	130 (19)	131 (21)	128 (16)	0.274
Diastolic blood pressure (mmHg)	77 (11)	78 (12)	76 (10)	0.228
Paroxysmal atrial fibrillation, n (%)	135 (62.2)	70 (63.6)	65 (60.7)	0.765
Persistent atrial fibrillation, n (%)	27 (12.5)	10 (9.1)	17 (15.9)	0.134
Permanent atrial fibrillation, n (%)	55 (25.3)	30 (27.3)	25 (23.4)	0.53
Coronary artery disease, n (%)	65 (30)	28 (25.5)	37 (34.6)	0.13
Hypertension, n (%)	128 (59)	87 (79.1)	81 (75.7)	0.549
Diabetes, n (%)	53 (24.4)	29 (26.4)	24 (22.4)	0.493
Chronic heart failure, n (%)	75 (34.6)	35 (32.8)	40 (37.4)	0.478
Stroke/TIA history, n (%)	24 (11.1)	8 (7.3)	16 (14.9)	0.071
CHA_2_DS_2_-VA Score, median and range	3 (2–4)	3 (2–4)	3 (2–4)	0.897
HAS-BLED Score, median and range	2 (1–2	2 (2–2)	2 (1–2)	0.702
**COPD, n (%)**	24 (11,1)	16 (14,5)	6 (5,6)	**0.029**
**Active or former smoking, n (%)**	91 (41.9)	55 (50)	36 (33.6)	**0.014**
≥2 cardiovascular risk factors, n (%)	172 (79.3)	90 (81.8)	82 (76.6)	0.35
≥3 cardiovascular risk factors, n (%)	101 (46.5)	52 (47.3)	49 (45.8)	0.83
**Charlson Comorbidity Index, median and range**	3 (1–5)	4 (3–8)	2 (0–3)	**<0.001**
Creatinine (mg/dL)	1.00 (0.84–1.24)	1.00 (0.83–1.25)	1.00 (0.85–1.21)	0.963
**NT-proBNP (pg/mL)**	1135 (244–3171)	703 (210–2222)	1549 (850–4084)	**0.010**
BNP (pg/mL)	209 (90–434)	129 (66–451)	220 (91–432)	0.463
LAA thrombi, n (%)	6 (2.8)	1 (0.9)	5 (4.7)	0.089
Major bleeding, n (%)	13 (6)	9 (8.2)	4 (3.7)	0.168
**Pulmonary vein isolation, n (%)**	25 (11.5)	2 (1,8)	23 (21.5)	**<0.001**
**Electrical cardioversion, n (%)**	55 (25.3)	18 (16.4)	37 (34.6)	**0.002**
**Antiarrhythmic drugs #, n (%)**	63 (29)	16 (14.5)	47 (44)	**<0.001**
VKA, n (%)	20 (9.3)	10 (9)	10 (9.3)	0.965
**LMWH, n (%)**	18 (8.3)	17 (15.9)	1 (0.9)	**<0.001**
DOAC, n (%)	143 (65.9)	68 (61.8)	75 (70.1)	0.759

# excluding beta blockers. Abbreviations: BMI—body mass index; BNP—B-type natriuretic peptide; COPD—chronic obstructive pulmonary disease; DOAC—direct oral anticoagulants; LAA—left atrial appendage; LMWH—low-molecular-weight heparins; NT-proBNP—N-terminal pro B-type natriuretic peptide; TIA—transient ischemic attack; VKA—vitamin K antagonists. Bold variables have a statistically significant value.

**Table 2:** Echocardiographic parameters in the study and control group.

Variable	All Patients n = 217	Cancer Patients n = 110	Control Group n = 107	*p* Value
LVEDV (mL)	119 (55)	107 (41)	132 (64)	0.059
**LVESV (mL)**	60 (44)	51 (33)	70 (52)	**0.032**
**LVIDd (mm)**	50 (8)	49 (7)	52 (9)	**0.018**
LVIDs (mm)	35 (9)	33 (8)	36 (10)	0.302
IVS (mm)	11 (2)	12 (2)	11 (2)	0.629
PW (mm)	11 (2)	11 (2)	10 (2)	0.151
**LA diameter (mm)**	45 (7)	43 (6)	47 (7)	**0.015**
**LA diameter > 40 mm (n, %)**	111 (51%)	48 (44%)	63 (59%)	**0.020**
LAA (cm^2^)	30 (10)	29 (9)	30 (11)	0.701
LAA > 20 cm^2^ (n, %)	58 (27%)	32 (29%)	26 (25%)	0.446
**LA volume (mL)**	102 (54)	90 (31)	114 (66)	**0.043**
LAVI (mL/m^2^)	53 (26)	49 (19)	58 (31)	0.087
LAVI > 34 mL/m^2^ (n, %)	93 (43%)	45 (41%)	48 (45%)	0.514
RAA (cm^2^)	25 (8)	24 (7)	25 (9)	0.704
**RAA > 18 cm^2^ (n, %)**	49 (23%)	16 (15%)	33 (31%)	**0.004**
**LVEF (%)**	53 (11)	54 (9)	51 (12)	**0.025**
**LV GLS (%)**	−15 (4)	−16 (4)	−13 (3)	**0.016**

Abbreviations: IVS—interventricular septum; LA—left atrial; LAA—left atrial area; LAVI—left atrial volume index; LV—left ventricle; LV GLS—LV global longitudinal strain; LVEDV—LV end-diastolic volume; LVEF—LV ejection fraction; LVESV—LV end-systolic volume; LVIDd—LV internal diameter in diastole; LVIDs—LV internal diameter in systole; PW—posterior wall; RAA—right atrial area. Bold variables have a statistically significant value.

## Data Availability

The original results presented in the study are included in the article; further questions can be directed to the corresponding author.

## Abbreviations

AF	atrial fibrillation
LV	left ventricular
CVRF	cardiovascular risk factors
LDL	low-density lipoprotein
VKA	vitamin K antagonists
LMWH	low-molecular-weight heparin
CRNMB	clinically relevant non-major bleeding
CCI	Charlson Comorbidity Index
DOAC	direct oral anticoagulant
LAA	left atrial appendage
COPD	chronic obstructive pulmonary disease
NTpro-BNP	N-terminal pro-B-type natriuretic peptide
BNP	B-type natriuretic peptide
BMI	body mass index
LA	left atrium
LVEF	left ventricular ejection
LV GLS	left global longitudinal strain
CA	catheter ablation
ICD	implantable cardioverter defibrillation
